# Hydrometallurgical recycling of steel grinding swarf *via* oxidative leaching using ferric chloride

**DOI:** 10.1039/d5ra06768e

**Published:** 2025-10-24

**Authors:** Thomas Ottink, Franco Garjulli, Max Lumetzberger, Denise C. R. Espinosa, Martina Petranikova

**Affiliations:** a Department of Chemistry and Chemical Engineering, Chalmers University of Technology Kemivägen 4 41296 Gothenburg Sweden ottink@chalmers.se; b Chemical Engineering Department, Polytechnic School, University of São Paulo Rua do Lago, 250 São Paulo Brazil; c Anferra AB Medicinaregatan 8a 41390 Gothenburg Sweden

## Abstract

Grinding swarf is a hazardous waste generated in hundreds of thousands of tons and currently has limited options for recycling. It is an environmental and economic burden for the manufacturing industry and new recycling processes are necessary for sustainable waste management. Ferric chloride (FeCl_3_) is an oxidant which can be used to extract metals from steel scrap to produce ferrous chloride (FeCl_2_) solutions. This was applied for recycling of grinding swarf containing 64% mostly metallic Fe by dissolving it in concentrated FeCl_3_. Optimization of leaching conditions showed that up to 94% of Fe was recovered as FeCl_2_ within 1 h of leaching with FeCl_3_, but that reaction temperature was difficult to control due to highly exothermic reactions. In contrast, classical leaching with hydrochloric acid only recovered 41% Fe from swarf in 2 h and forms large volumes of flammable H_2_. This improvement in efficiency was attributed to the leaching mechanisms of FeCl_3_ which are kinetically superior and capable of circumventing lubricant components which otherwise protect the steel surface. These findings contribute to the development of a safe recycling process for valorisation of grinding swarf. Production of iron chloride solutions with applications in water treatment promotes recycling and reduces incineration and landfilling of this waste.

## Abstract 2

Recovery of iron from waste grinding swarf from manufacturing by production of iron chloride solutions with applications in sustainability areas.

## Graphical abstract

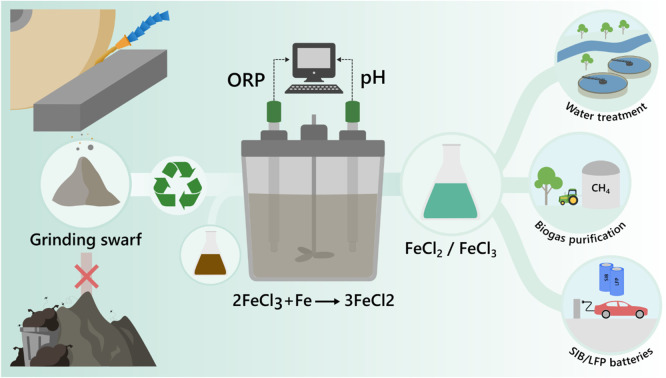

## 1 Introduction

Machining swarf is a waste generated in the steel and manufacturing industries when removing excess metal from a workpiece by machining operations such as turning, milling and grinding.^1^ Swarf is a collective term for metal chips stemming from these processes, which are often mixed with lubricants and particles from the machining tool. Swarf from grinding processes is unique due to its small particle size and large surface area which enables it to absorb more machining fluids.^2^ This leads to higher amounts of contaminants in the material which makes waste management difficult.^3^ A lack of feasible recycling options means that large amounts are today incinerated and/or landfilled.

Many countries in and outside the EU have restrictions on total organic carbon (TOC) and landfilling of flammable waste.^4^ Grinding swarf with oil or emulsion type lubricants typically fall under this category since they are known to self-ignite.^5^ Destruction of the organic fraction is therefore required, and incineration of grinding swarf with landfilling of ashes is today widespread. This is both a waste of energy and materials, and costly for the waste producers with disposal fees up to 1200 € per ton.^1^

The automotive and bearing industries have been identified as two main producers of grinding swarf, but most metal workshops can be expected to generate some amount. Estimating volumes globally between these many actors is impossible but numbers between 130 and 250 kton were reported by German industry alone between 1990 and 2000.^1^ and these numbers can be expected to have doubled with the past growth in global steel production.^6^

Many attempts have been made to extrude or wash out cutting fluids from swarf to facilitate recycling in steelmaking.^2,7–10^ While such collaborations between manufacturers, steel industry and third-party recyclers are occasionally successful, several obstacles have been identified by interviewing industrial actors. The inherent fire risk associated with swarf makes it difficult to transport and stockpile, and volumes from individual manufacturing sites are comparably low for the mill to process which requires advanced logistics and collection from multiple waste sources. Steel producers also voice concerns about contaminants in mixed swarf which could potentially poison their steel product, and the risk of flames and explosions when processing oily swarf.^2^ Moreover, the recycling yield is low in scrap processing due to the degree of oxidation and particulate nature of the swarf, causing it to combust easily and end up in slag or flue dusts. These drawbacks rarely justify the risk and economic gain from including grinding swarf in the melt.

The need for a specialised treatment for these smaller steel-waste streams was thus identified and a hydrometallurgical approach was proposed in Ottink (2024).^11^ Hydrometallurgy is more economical and energy efficient in small scale than pyrometallurgical processes and is especially well suited for recovery of metals from low grade ores and waste streams. In previous work, grinding swarf was leached with hydrochloric acid (HCl) to form an iron chloride solution which was purified to commercial EU standards (EN 888:2023) by precipitating and filtering out alloying elements and lubricant oils. Ferrous (FeCl_2_) and ferric (FeCl_3_) chloride solutions have use in water treatment as coagulants, in etching of electronic components, biogas upgrading, as precursors for battery materials, *etc.*^12–15^ Converting waste into products that serve these downstream applications creates strong economic incentives for recycling. This can make both the steel and water industry more sustainable as virgin magnetite is still a main feedstock in the production of coagulants. Environmental benefits from hydrometallurgical recycling of grinding swarf over incineration and recycling in steelmaking furnaces were recently verified by an independent third party.^16^

A potential hazard when dissolving metallic waste in HCl batchwise is that large amounts of intermittent, explosive hydrogen gas (H_2_) are formed, in total around 30 kg per ton of swarf.^11^ While this fits well with current efforts to produce green H_2_ in the steel and chemical industries, it is questionable whether capture in these small volumes is economically feasible. A typical large workshop can produce one thousand ton grinding swarf annually which would generate up to thirty tons of H_2_. Unless direct local utilization is possible, these volumes are insignificant relative to the current global demand of one hundred million tons.^17^

An alternative to acidic leaching is to use another oxidizing agent such as FeCl_3_. This has been investigated extensively in extraction of copper, zinc, lead, nickel, *etc.* from sulphide minerals.^18–21^ Here, the ferric ion acts either directly as an oxidising agent or as a chloride carrier for chlorine gas formation to oxidise sulphur and lixiviate the desired metal. Another use is the dissolution of noble metallic compounds such as gold and copper which are mostly insoluble in mineral acids. In these cases the ferric ion oxidises and chlorine helps with formation of soluble chloride complexes.^22,23^ The latter mechanism can also be used in dissolution of steel scrap and has been proposed as an alternative method to dissolve metals with reduced H_2_ formation.^24,25^ Application of FeCl_3_ to recover metals from grinding swarf has however not been reported. The aim of this work was to investigate oxidative leaching of swarf with FeCl_3_ to produce iron chloride solutions. This will lay the foundation for a more reliable, sustainable and safe hydrometallurgical recycling scheme for this type of waste.

## 2 Theory

The desired reaction when leaching swarf with FeCl_3_ is the oxidation of metallic Fe by Fe^3+^*via*eqn (1).

1 Fe + 2Fe^3+^ ⇌ 3Fe^2+^

Reaction between two species with different oxidation states of the same metal that form a species with an intermediate oxidation state is called comproportionation.^26^ This mechanism can be useful for performing reactions without introducing any foreign substances into a system. Comproportionation is best described by Frost diagrams and a diagram for Fe in acidic solutions at pH 0 is given in the supplementary material Fig. S1. In the diagrams, oxidation states are compared by their relative stability *via* the electrochemical Gibbs free energy in eqn (2).

2 Δ*G* = −*nFE*°

where *n* is the number of electrons transferred, *F* is the Faraday constant and *E*° is the standard potential for reduction of Fe^2+^, Fe^3+^, *etc.* to the ground state. A lower point indicates higher stability and the formation since Fe^2+^ is located below Fe^3+^ and Fe, reaction (1) is feasible.

Ideally, leaching of metallic Fe with FeCl_3_ doesn't form any byproducts according to eqn (1). However, Fe^3+^ can undergo side reactions in aqueous chloride solutions which may interfere. Chlorinated complexes of Fe^3+^ can form by replacing water with Cl^−^ in its coordination sphere *via*eqn (3).^27,28^

3 Fe^3+^ + *x*Cl^−^ ⇌ FeCl_*x*_^(3−*x*)+^

The degree of chlorination depends on chemical equilibria and increases with [Cl^−^]. Possible species include FeCl^2+^, FeCl_2_^+^, FeCl_3_^0^ and FeCl_4_^−^. Besides formation of chloride complexes, Fe^3+^ also has a strong tendency to hydrolyse *via*eqn (4).

4 Fe^3+^ + *x*H_2_O ⇌ Fe(OH)_*x*_^(3−*x*)+^ + *x*H^+^

This reaction is influenced by both pH and temperature. An increase in [H^+^] suppresses hydrolysis while high temperatures make it more thermodynamically and kinetically favourable. Because Fe^3+^ hydrolyses easily, the pH of concentrated FeCl_3_ solutions is usually <1.^28^ Both Fe^3+^ and its chloride complexes can form a myriad of aqueous hydrolysis products including FeOH^2+^, Fe(OH)_2_^+^, FeCl(OH)^+^, FeCl(OH)_2_^0^ and solid Fe(OH)_3_. Besides these commonly reported forms, dimeric, trimeric and polymeric hydroxide derivatives of Fe^3+^ have also been isolated.^29^

Analogously, Fe^2+^ can also react with Cl and H_2_O *via* reactions similar to eqn (3) and (4) but it hydrolyses to a lesser extent making it more stable even in less acidic conditions of pH 3–4.^30–32^ For clarity, the collection of all aqueous species of Fe^2+^ and Fe^3+^ are hereafter referred to as Fe(ii) and Fe(iii) respectively.

The most problematic side reaction in terms of leaching is hydrolysis since this releases H^+^ into the solution which can react irreversibly with metallic Fe *via*eqn (5).

5 Fe + 2H^+^ → Fe^2+^ + H_2_

This reaction is spontaneous with Δ*G*° = −44.8 kJ mol^−1^ and produces Fe^2+^ but at the expense of H_2_ formation and potential precipitation of Fe(iii) by hydrolysis *via*eqn (6).

6 Fe^3+^ + 2H_2_O → FeO(OH) + 3H^+^

At 25 °C, Δ*G*° = 1 kJ mol^−1^ for this reaction but the value decreases with increasing temperature. While this can result in a net solubilization of Fe (3 : 2 Fe^2+^ : FeO(OH) formation), the reagent is lost in the form of hydrated akaganeite (β-FeO(OH)·H_2_O) when leaching in chloride media.^33^

## 3 Experimental

Grinding swarf was provided by Scania AB Sweden and was received as filtered. The sample originated from grinding of cast iron camshafts using mineral oil based semi-synthetic cutting fluids (Quakercool 3750 BFF) and cubic boron nitride (CBN) abrasives. To homogenize the material, approximately 200 g of swarf was placed in a sample splitter (Gilson Spinning Riffler) and divided into 20 portions of 8–10 g each. Samples were randomized completely for chemical analysis and leaching experiments.

### 3.1 Characterization

Determination of solid and liquid fractions in the swarf was done by washing 500 g swarf in 2 L ethanol in a beaker while mixing for 15 minutes. Washed swarf was filtered and rinsed with an additional 1 L ethanol before drying in an evaporating dish in a fume hood at 21 °C for 48 h until further weight loss by solvent evaporation was negligible. Liquid contents were then estimated gravimetrically by weighing the swarf before and after washing. Further chemical analysis of solid materials was done by digestion in aqua regia and inductively coupled optical emission spectroscopy (ICP-OES), electron microscopy (SEM), and X-ray diffraction (XRD).

#### 3.1.1 Digestion and ICP-OES analysis

Contents of digestible metals in the swarf were determined by ICP-OES (iCAP™ PRO XP, Thermo Fisher). Around 0.5 g material was dissolved in triplicate in 30 mL aqua regia prepared in a 1 : 3 ratio of HNO_3_ (69%, Merck, Suprapur) and HCl (37%, Sigma Aldrich, ACS reagent). The mixture was heated to 80 °C for 4 h and after digestion, the solution was passed through filter paper (Whatman 1) and diluted with Milli-Q water in 50 mL volumetric flasks. Aliquots from each flask were filtered again using 0.45 μm syringe filters and diluted further with 0.1 M HCl before ICP-OES analysis. Calibration of the ICP-OES was done using standards with elemental concentrations between 0.5 and 20 ppm. These were prepared from 1000 ppm single element solutions (Inorganic Ventures) by dilution in 0.1 M HCl.

#### 3.1.2 Electron microscopy

Swarf was further analysed using scanning electron microscopy coupled with energy-dispersive spectroscopy (SEM-EDS) to understand the microscopic structure of the material and study local oxidation. A small portion of the sample was placed on a stub with graphite adhesive tape. The sample was examined using a Phenom ProX™ equipped with BSD detectors at a voltage of 15 kV. The EDS analysis employed the ZAF quantification method.

#### 3.1.3 XRD analysis

Phase compositions of swarf and other solids were studied qualitatively using a diffractometer (Bruker D8 Advance) with a Cu source of wavelength 1.5406 Å. Analysis was done in a 2*θ* range of 10–80° and peaks in the diffractograms were correlated to crystalline phases by comparison with the International Centre for Diffraction Data (ICDD) database.

### 3.2 Leaching experiments

Leaching experiments were conducted in a 100 mL jacketed glass reactor. Temperature control was achieved by circulating hot water from a thermostatic bath through the outer layer of the reactor. The setup was fitted with a combined pH glass electrode (Unitrode with Pt1000, Metrohm) and redox electrode (Pt ring electrode, Metrohm) to simultaneously measure temperature, pH and oxidation–reduction potential (ORP). Solutions were stirred electrically with a polypropylene propeller operating at 1800 rpm. Both electrodes and stirrer were connected to an automatic titrator (Titrando 905) and controlled *via* Tiamo™ software.

Unless stated, 35 mL of 32.5 wt% FeCl_3_ solution was added to the reactor in each experiment. This solution was prepared with Milli-Q water and solid FeCl_3_ (≥97%, Sigma Aldrich) in an E-flask while externally cooling with water. The reactor was preheated and once the desired leaching temperature was reached, swarf was added and leaching proceeded for a designated amount of time.

After reaching the time limit, leachate was filtered (Whatman, Grade 1), and the filter cake was washed with 10–20 mL Milli-Q water until any colour from the solution disappeared from the paper. The aqueous phase was weighed, and an aliquot was diluted with 0.1 M hydrochloric acid before analysis with ICP-OES. Since Fe and some alloying elements were present in both FeCl_3_ reagent and in the swarf, two interpretations of leaching efficiency could be defined. Firstly, the amount of metal M extracted from the solids (% *E*_s_) according to eqn (7).7
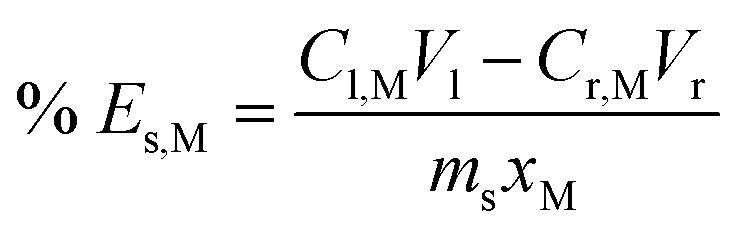


And secondly, the total efficiency (% *E*_tot_) for metal M was defined by eqn (8).8
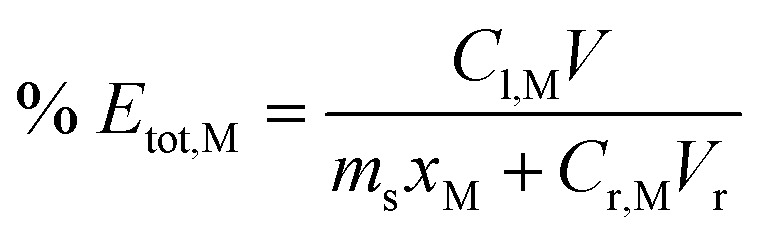


In each equation s, l, and r denote solid, leachate and reagent respectively, *C*_i,M_ is the concentration of M in solution i with volume *V*_i_, and *m*_s_ is the mass of solids with mass fraction *x*_M_ of metal M. Some precaution is advised when interpreting data using these definitions since eqn (7) is based on the distribution of a metal between the aqueous and solid phase and does not account for whether some iron reagent could have precipitated in the process. Eqn (8) on the other hand describes the overall atom efficiency but gives little indication of how much has been extracted from the solids.

### 3.3 Design of experiments

Design of experiments (DOE) is an experimental methodology where the effects of multiple variables on a given response can be evaluated simultaneously.^34^ In this study, a face centred central composite (CCD) design was used to determine the influence of temperature (*T*, coded *x*_1_), time (*t*, coded *x*_2_), and ratio of reagent volume to solids (L/S ratio, coded *x*_3_) on extraction efficiency % *E*_s_ as defined by eqn (7). A 2^3^ factorial design formed the basis for the experiments, and three replicates of the centre point were included for estimation of the pure error for the whole experimental range. Axial runs at *α* = 1 were included to account for any quadratic effects in the following regression modelling. A face centred design was selected on the basis that extremes in the variable levels should be avoided. All experiments were run in random order to account for experimenter bias.

Interpretation of DOE results was done *via* regression modelling, analysis of variance (ANOVA) and response surface methodology. A model including first order effects and interactions and second order effects as described by eqn (9) was fitted to experimental data *via* the least square method.

9 % *E*_s,M_ = *β*_0_ + *β*_1_*x*_1_ + *β*_2_*x*_2_ + *β*_3_*x*_3_ + *β*_12_*x*_1_*x*_2_ + *β*_13_*x*_1_*x*_3_ + *β*_23_*x*_2_*x*_3_ + *β*_11_*x*_1_^2^ + *β*_22_*x*_2_^2^ + *β*_33_*x*_3_^2^ + *σ*

Optimization to reduce overfitting was then done by residual analysis and stepwise removal of model terms until a minimum lack of fit and maximum adjusted *R*^2^ value were achieved.

### 3.4 Iron speciation techniques

Leaching performance was evaluated further by speciation of Fe as a complement to ICP-OES analysis. Two different methods were employed: (1) complexation of Fe^2+^ with phenanthroline and subsequent analysis with UV/vis spectroscopy,^35^ and (2) direct redox potential measurement in the leaching slurry. The former was found to be unsatisfactory and is only presented in the supplementary materials. Variability in [Fe(ii)] and total [Fe] readings were high with this method resulting in unrealistic leaching efficiencies >100% and in some cases higher [Fe(ii)] than total [Fe]. A possible source of this error was the many steps involved in sample preparation after a leaching experiment. Measurement of redox potentials in concentrated iron chloride solutions was however found to be a surprisingly good indicator of Fe(iii) content in Fe(ii). This required calibration of the electrode by titrating FeCl_2_ with FeCl_3_ solutions of known concentrations at various temperatures.

#### 3.4.1 Electrode calibration for redox potential measurements

The same setup used in leaching experiments was utilized to titrate FeCl_2_ solutions with FeCl_3_ while measuring ORP and pH. A FeCl_2_ solution containing 200 g Fe per L was prepared by dissolving 100 g Fe powder (≥99%, Sigma Aldrich) in 298 mL 37% HCl in a covered E-flask while heating to 80 °C using a hot plate. More HCl was added dropwise if any Fe powder remained and a pH value of >3 was measured, while water was added if FeCl_2_ started crystallizing during the preparation. This is necessary since some of the reagent tends to evaporate when heated. The solution was transferred to a volumetric flask, made up to 500 mL and stored sealed with a stopper and parafilm wrapping to minimize oxidation. A similar FeCl_3_ solution containing 200 g Fe per L was prepared using the same procedure as described in Section 3.2. The final pH of FeCl_2_ was adjusted to −0.5 with HCl to match the value measured in FeCl_3_.

The FeCl_3_ solution was transferred to a bottle equipped with a dosing device (10 mL 800 Dosino, Metrohm) connected to the automatic titrator. In a measuring cylinder, 50 mL of FeCl_2_ was weighed and added to the reactor. The solution was heated to 20, 40 or 60 °C and purged with N_2_ before and during titration. Around 0.1 g Fe powder was added to reduce any FeCl_3_ and after 5 minutes of mixing, a magnet was used to remove the powder. A colour transition from green to clear blue and a stable measured ORP between −300 to −250 mV confirmed that the reduction was complete. The reactor was then resealed and titration commenced.

## 4 Results and discussion

### 4.1 Characterization

**Fig. 1:**
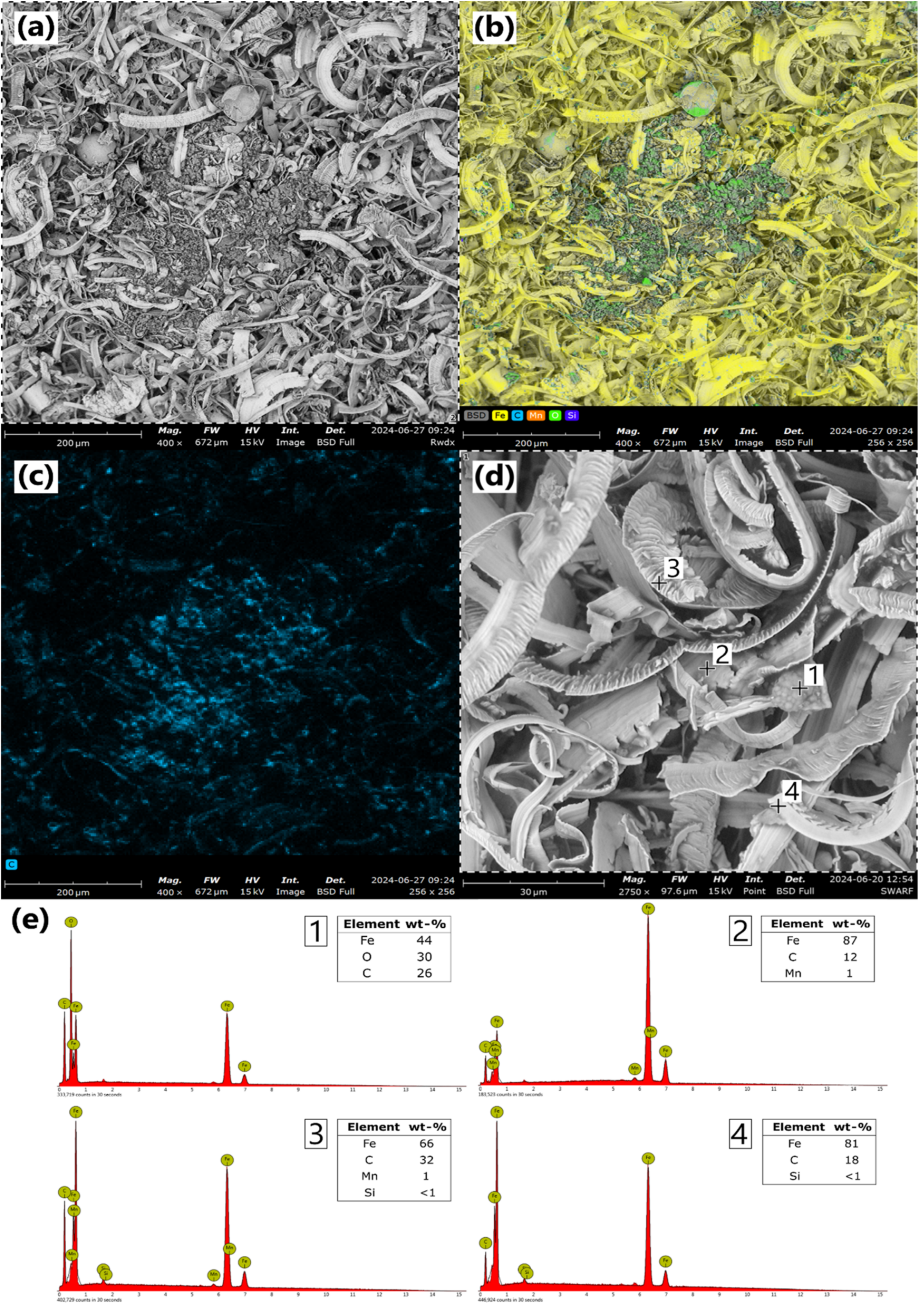
Backscatter electron SEM image of an oxide cluster at 400× magnification (a) with a corresponding EDS elemental map (b), and carbon mapping (c). A typical image of the swarf shavings at 2750× magnification (d) with corresponding EDS spot analysis at selected locations (e).

The grinding swarf investigated in this study had a fluffy, carpet-like structure after separation from grinding fluids *via* vacuum filtration. Most of the swarf had a steel grey colour but some local oxidation had also taken place after storing the sample in a sealed container for several weeks. A closer look at the morphology of the swarf by given in SEM images in Fig. 1.

The backscatter SEM images show that most of the grinding swarf is composed of longer sickle-shaped shavings which explain the cohesive nature of the material. Besides these oblong steel particles, iron oxide clusters were also found of which a typical example is shown in Fig. 1a and b. The sample was difficult to flatten and as such the topography was uneven leading to some undesirable contrast and shading effects. Nonetheless, the elemental mapping shows that smaller growths and larger spheres and clusters of oxides were found throughout the swarf. Some corrosion can be expected in air since the swarf has a large surface area and is covered in water-based lubricant. Organic corrosion inhibitors in the cutting fluids can however slow this process.^36^ Formation of the larger oxide clusters is the result of an exothermic corrosion reactions where oxidation of the steel releases heat and in turn promotes further metal oxidation. If left uncontrolled, this can eventually lead to a thermal runaway and combustion of metals and oils.^5^ Auto-ignition of swarf is most common with emulsion type lubricants according to interviews with industrial actors.

Fig. 1d shows shavings at higher magnification and spot analysis was performed with spectra given in Fig. 1e. In spot 2, an oxide lump was studied, and the molar ratio Fe/O ≈ 1.5 suggests that it was composed of iron oxide hydroxide (FeO(OH)) rather than hematite or magnetite. Spots 3, 4 and 5 focused on the metallic shavings and as expected, these were mostly metallic Fe, but a high carbon content was also measured. This signal came from organic carbon in the lubricants which cover the metal surfaces and are easily detected since the electrons have a limited penetration depth. The signal for C was stronger for the folded shaving at point 4 which has a higher surface area and can accumulate more lubricant.

Surprisingly, higher carbon contents were also detected in the corroded areas according to Fig. 1c. This suggests that the cutting fluids had a greater tendency to accumulate at the oxide surfaces. The lubricant in this sample was a semi-synthetic emulsion and was expected to contain amphiphilic molecules including emulsifiers, corrosion inhibitors, defoamers, *etc.*^36^ These substances can adsorb depending on the polarity of the surface and molecule. Since iron oxide is a mixture of strongly electronegative oxygen and electrophilic iron, it can be expected to have a greater affinity for the polar organic molecules than the metallic surfaces have. In terms of corrosion, a noteworthy consequence of this is that the lubricant's metallic surface coverage decreases with oxidation which can facilitate further corrosion.

The grinding swarf's elemental composition and contents of cutting fluids and other solids are reported in Table 1. Digestible metals in the steel fraction were analysed by aqua regia dissolution followed by ICP-OES and show that the swarf was a suitable candidate for production of FeCl_2_ based on the high Fe content and few alloying elements. Of the more problematic metals in water treatment, only Cr was found in higher concentrations and only traces of Ni and Cu were detected. Besides Fe, the swarf also contained comparably high amounts of cutting fluids which included both water and organic substances. Other unidentified solids may comprise oxygen based on SEM images in Fig. 1, as well as inorganic carbon from the steel and ceramic grinding wheel components (CBN) which are insoluble in aqua regia.

**Table 1:** Composition of the grinding swarf determined using ICP-OES after aqua regia digestion for metals, and solvent washing and gravimetry for cutting fluids. Other substances may include grinding wheel abrasives and binders, inorganic carbon from the steel and oxygen from corrosion

Compound	Wt%	Compound	Wt%
Fe	64.03 ± 1.54	Mo	0.04 ± 0.01
Mn	0.94 ± 0.01	Al	0.02 ± 0.01
Cr	0.18 ± <0.01	Co	<0.01
Si	0.17 ± 0.03	Zn	<0.01
V	0.06 ± 0.01	Cutting fluids	16.0
Ni	0.04 ± 0.01	Other solids	18.5
Cu	0.04 ± 0.01		

### 4.2 Redox potentials at different Fe(iii) and Fe(ii) ratios

**Fig. 2:**
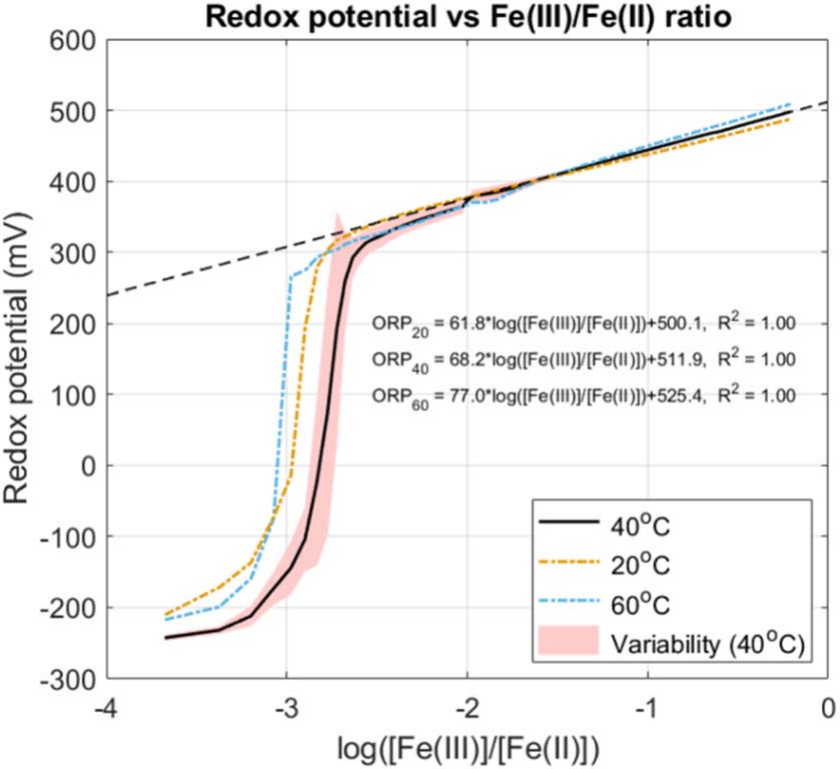
Measurement of redox potential for [Fe(iii)]/[Fe(ii)] ratios at 20 °C, 40 °C and 60 °C and constant total [Fe] = 200 g L^−1^. Experiments at 40 °C were performed in triplicate and the line represents average values with standard deviations visualized by the shaded area. Linear regression was done for all instances in the range log([Fe(iii)]/[Fe(ii)]) > −2.

Results from titration of synthetic FeCl_2_ with FeCl_3_ for speciation of Fe at three different temperatures are shown in Fig. 2. Redox potentials are unadjusted measurements from the same electrode used in leaching and are presented as a function of the logarithmic concentration ratio between Fe(iii) and Fe(ii) to better highlight ORP changes in early FeCl_3_ additions. Between practically having no Fe(iii) to log([Fe(iii)]/[Fe(ii)]) = −2 (1% Fe(iii)), the ORP increased from <−200 mV to 350 mV and the solution's colour changed from blue to dark brown. Around the inflection point the ORP fluctuated as shown by variability in the data for the 40 °C case, and as a result estimating [Fe(iii)]/[Fe(ii)] accurately might be difficult. However, as more FeCl_3_ was added ORP measurements became remarkably consistent and took on a linear relationship *versus* log([Fe(iii)]/[Fe(ii)]). Slightly higher ORP was observed at 60 °C and slightly lower at 20 °C.

Even small amounts of Fe(iii) have a large influence on the overall ORP of the system. The Nernst equation for estimating ORP for this specific case is given by eqn (10).10
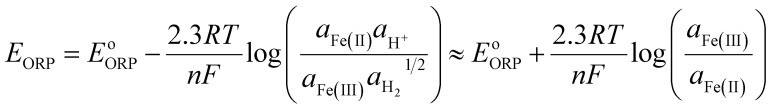


It can be assumed that *a*_H_2__ = 1 and that *a*_H^+^_ remains constant during the titration based on an average voltage drop of 10 mV measured by the pH electrode during experiments. Therefore, changes in *E*_ORP_ are dominated by the activity ratio of Fe(iii) and Fe(ii) species. What Fig. 2 suggests is that the relative activity of Fe(iii) increases dramatically when 0–1% is added to the FeCl_2_ and then proceeds to increase logarithmically. A main implication of this is that even small amounts of Fe(iii) produce oxidative conditions and can be a good leaching agent for metallic Fe regardless of its concentration. Additionally, the linear relations show that redox potentials can be fine-tuned in these types of solutions by controlling Fe(iii)/Fe(ii) ratios. These results may therefore also be of interest for other redox-dependent systems such as leaching of sulphide minerals.^37^

Fig. 2 can be a useful tool for Fe(iii)/Fe(ii) systems but some precaution is advised when interpreting ORP data. Normally the accuracy of redox electrodes is around ±10 mV which means that other electrodes can give slightly different values.^38^ The graphs are only valid for 200 g Fe per L and any deviations or presence of impurities may affect ORP according to eqn (10). Finally, temperature can also influence ORP in unforeseeable ways and these effects were not compensated by the titrator. Regardless, detection of Fe(iii) and rough estimation of concentration ratios using ORP was possible and was used as a tool in following leaching tests.

### 4.3 Experimental design (TO)

Experimental design conditions and selected responses are shown in Table 1. Experiments 1–8 (standard order) represent the base factorial experiments, 9–11 are centre point replicates, and 12–17 experiments on the face of the design.

A general conclusion that could be drawn from the data was that it should be possible to find an optimal L/S since no Fe(iii) remained at the lower extreme and 3–16% was left at the high level. Theoretically, around 70% Fe can be extracted with 6 mL g^−1^, 90% with 8 mL g^−1^ and 115% with 10 mL g^−1^ based purely on eqn (1) and responses for Fe were below these limits. Table 2 also shows that responses for Mn conformed well to the theoretical limits which indicated that dissolution of the metallic steel was successful. Efficiencies for Cr were high when residual Fe(iii) was present at pH < 3.5, and low when all Fe(iii) had been reduced in combination with pH ≥ 3.5. This was due to hydrolysis and precipitation at less acidic conditions according to eqn (11).^39^

11 Cr^3+^ + 3H_2_O ⇌ Cr(OH)_3_(s) + 3H^+^

**Table 2:** Experimental design conditions and solid leaching efficiency (% *E*_s,M_) responses for Fe, Mn and Cr. Final ORP and pH values measured in the leaching slurry are also given as well as an estimate of remaining Fe(iii) *via* speciation based ORP from Fig. 2. Other conditions: [FeCl_3_] = 32.5 wt%, stirring at 1500 rpm

Standard order	Random order	Variables			Responses (% *E*_s_)			Potentials		[Fe(iii)]/[Fe(ii)] (%)
		*T* (°C)	*t* (min)	L/S (mL g^−1^)	Fe	Mn	Cr	ORP	pH	
1	1	20	15	6	23	61	14	−480	3.7	<0.1
2	5	60	15	6	14	48	0	−494	3.8	<0.1
3	11	20	105	6	58	70	0	−487	4.3	<0.1
4	10	60	105	6	64	73	0	−439	3.8	<0.1
5	7	20	15	10	85	100	100	410	1.7	3.5
6	17	60	15	10	78	92	62	437	0.8	7.2
7	6	20	105	10	94	100	95	406	1.9	3.0
8	9	60	105	10	93	100	80	441	0.7	8.0
9	12	40	60	8	86	89	0	−478	3.9	<0.1
10	4	40	60	8	70	84	0	−481	3.8	<0.1
11	14	40	60	8	76	94	0	−469	3.7	<0.1
12	15	20	60	8	67	84	36	−363	3.5	<0.1
13	8	60	60	8	72	85	0	−438	3.3	<0.1
14	13	40	15	8	83	92	25	−410	3.3	<0.1
15	16	40	105	8	90	94	0	−481	3.9	<0.1
16	3	40	60	6	60	63	0	−489	4.1	<0.1
17	2	40	60	10	64	100	92	457	0.8	15.7

Time clearly also played a role based on responses for Fe in experiments 1–4 in Table 2 which will be discussed further later. To draw more sound conclusions about the influence of different variables, regression modelling was done next.

#### 4.3.1 Regression and response surface modelling

A model based on eqn (9) was fitted to response data for Fe in Table 2. The full model exhibited low significance with a low *R*_adj_^2^ = 0.68 and high variability with *σ* = 12.5%. To improve the model, parameters with least significance were eliminated to reduce overfitting.^34^ This was done by eliminating *x*_1_*x*_3_, *x*_1_, *x*_1_*x*_2_ and *x*_1_^2^ stepwise and it was found that removal of *x*_1_*x*_3_, *x*_1_ and *x*_1_*x*_2_ positively affected predictability according to supplementary Table S1. The final model for data prediction is given in eqn (12). Effects from insignificant variables explain the experimental data variability (*σ* = ±10.6%).

12 % *E*_s,Fe_ = 79.2 + 13.2*x*_2_ + 19.5*x*_3_ − 7.8*x*_2_*x*_3_ − 8.0*x*_1_^2^ + 9.1*x*_2_^2^ − 18.2*x*_3_^2^ + *σ*

**Fig. 3:**
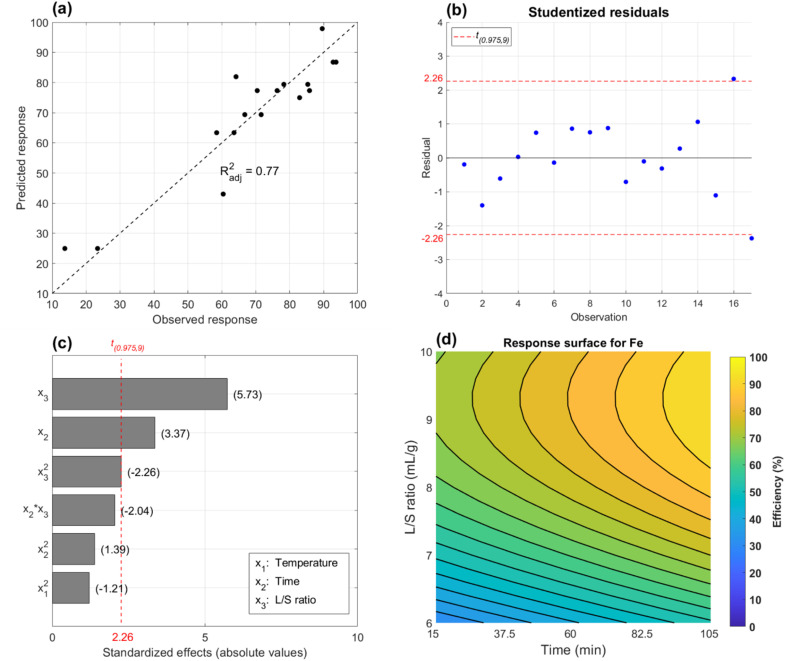
Regression model diagnostics and response surface for leaching of Fe. Predicted *versus* observed responses with adjusted *R*^2^ (a), standardized effects and significance of regression parameters (b) and studentized residuals *vs.* a *t*-distribution with 95% confidence (c) for the model in eqn (12). For the same model but including only significant parameters *x*_2_, *x*_3_ and *x*_3_^2^, a contour plot of the response surface (d).

Further regression model diagnostics and a response surface based on eqn (12) are shown in Fig. 3. According to the predicted value modelling in Fig. 3a, experimental data was acceptable with a final *R*^2^ = 0.85 and *R*_adj_^2^ = 0.77. Two borderline outliers were identified by residual analysis in Fig. 3b which stemmed from experiments 16 and 17 (standard order). No obvious problems were recorded during experimentation however a high amount of unreacted Fe(iii) was left in experiment 17 compared to experiments 5–8 with identical L/S = 10 mL g^−1^. A potential explanation was that sample 17 contained more oxides which are insoluble when leaching solely with Fe(iii). This was reasonable since it was shown that the swarf was heterogeneous with oxide clusters as seen in Fig. 1. Consequentially, model predictability is poorer at L/S extremes on the faces of the design.

A visual representation of regression parameter significance *versus* the t-distribution is given in the Pareto diagram in Fig. 3c. It was concluded that L/S ratio and time were most influential on Fe leaching while secondary L/S effects were also borderline significant. Temperature notably didn't have a significant effect on the leaching of Fe. The contour plot in Fig. 3d was used to visualize how significant parameters affected leaching. This surface was based on a further reduced regression model including only the three significant terms (*x*_2_, *x*_3_ and *x*_3_^2^), obtained by removing other terms in eqn (12). Optimal leaching conditions were found to be L/S = 9 mL g^−1^ and a leaching time >90 min to ensure complete dissolution of Fe with minimal reagent. The reason time is important is not obvious. According to final potentials data for experiments 1–4 in Table 2, no Fe(iii) was left in solution and pH > 3.5 in each experiment. Still, an efficiency increase of 40% for Fe was seen between 15 and 105 minutes of leaching. An attempt to explain this behaviour is made in the next section.

#### 4.3.2 Leaching time effects in centre point experiments

**Fig. 4:**
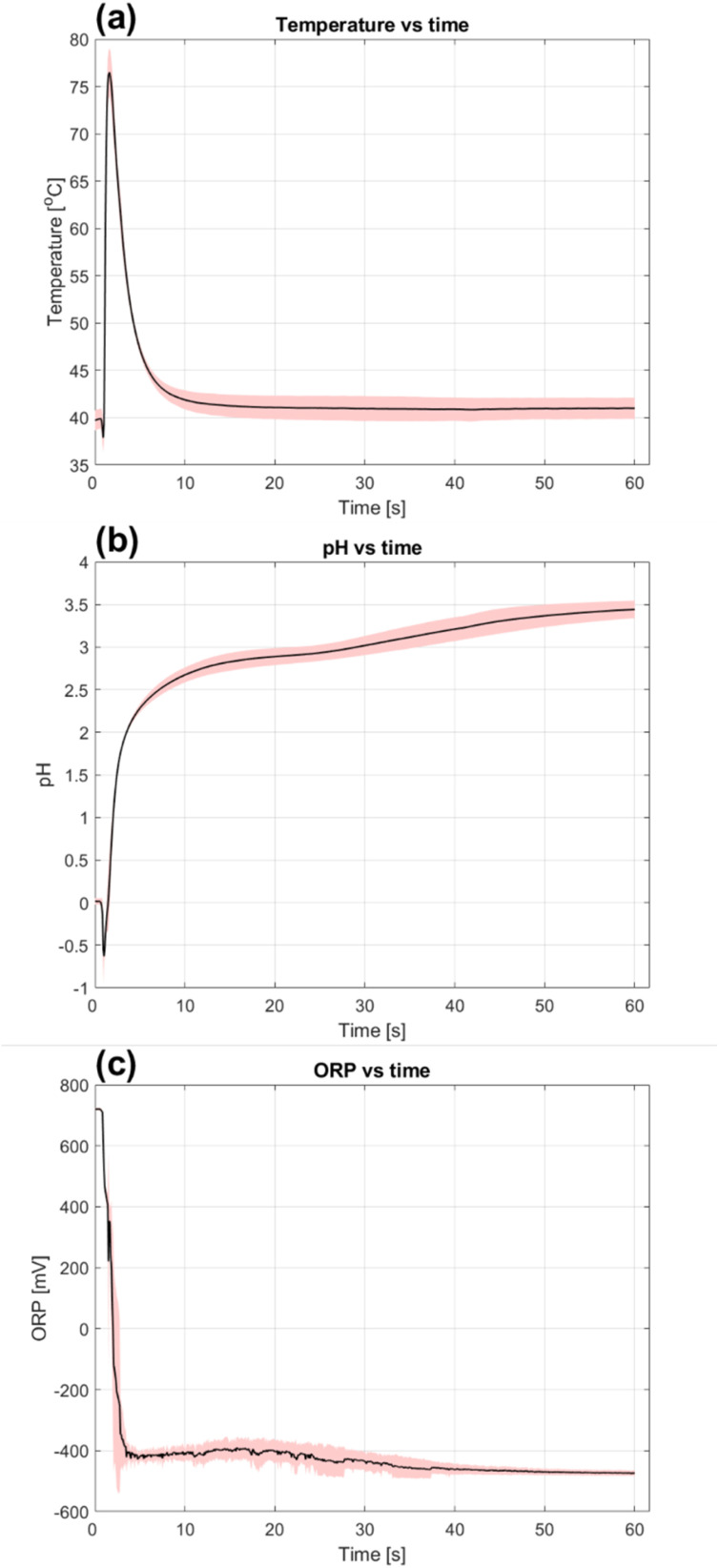
Average temperature (a), pH (b) and ORP (c) *versus* time in experimental design centre point experiments. Data variability is represented by the red shaded areas for each case.

Changes in the aqueous phase could be monitored carefully over time using electrodes. Average temperature, pH and ORP data and variability in DOE centre point experiments are shown in Fig. 4. These results were representative of trends observed in all DOE experiments.

A first observation that can be made in Fig. 4a is that there was a large temperature increase of 40 °C within the first five minutes despite externally cooling the reactor. This was caused by rapid reaction between the swarf and FeCl_3_ as seen by the sharp ORP drop from +700 mV (100% Fe(iii)) to −400 mV (<1% Fe(iii)) in Fig. 4c. Experimentally the reaction was also observed to be violent with almost instantaneous dissolution of the steel and significant sizzling and bubbling. The reaction between metallic Fe and FeCl_3_ is highly exothermic with Δ*H*_r_ = −161.4 kJ mol^−1^ and explains this heat development. A consequence was that temperature could not be properly controlled throughout experimentation and its effect on the initial reaction can be difficult to interpret. Longer term temperature effects on leaching should however still be interpretable since the system returned to the set point of 40 °C at 15 minutes but were still considered insignificant based on DOE results.

According to Fig. 4c, all Fe(iii) detectable by the ORP electrode disappeared in the first five minutes. Aqueous Fe(iii) could be consumed in two different ways, firstly *via* the desired reaction between metallic steel and FeCl_3_, and secondly *via* hydrolysis and unwanted precipitation. There is evidence of the latter in Fig. 4b which shows that the pH drops from an average 0 to −0.6 when swarf is added to the reactor. Rust coloured solids were also seen during experiments in the first minutes of reaction. In this case hydrolysis was induced by contact with the swarf, which has an alkaline nature due to amines and inorganic salts in the semi-synthetic cutting fluids.^36^ Simultaneously, heat developed by chemical reactions contributed to further hydrolysis. Previous work on synthesis of hematite from FeCl_3_ solutions show that Fe(iii) precipitates as akaganeite (β-FeO(OH)) *via*eqn (13) in FeCl_2_ solutions which can then further recrystallize to hematite (Fe_2_O_3_) *via*eqn (14) at >140 °C.^33,40,41^

13 Fe^3+^ + 2H_2_O ⇌ FeO(OH) + 3H^+^

14 2FeO(OH) + H_2_O ⇌ Fe_2_O_3_ + 3H^+^

These reactions are favoured by high temperatures and precipitation can be expected to have taken place near the swarf surface where heat development was prominent.

Although some reagent was precipitated, Fig. 4b also shows that H^+^ released by hydrolysis was quickly consumed again with a pH increase from −0.6 to 2.8 after 15 minutes of leaching. The acid could have been consumed either by dissolution of hydrolysis products or any remaining metallic Fe. If solid Fe(iii) was redissolved, no marked effect was noted on ORP according to Fig. 4c. This is however not surprising since aqueous Fe(iii) could have reacted quickly with remaining metals in the swarf as indicated by the initial rapid ORP decrease. A slight decrease in ORP from −400 mV to −470 between 5–60 minutes when the pH increased from 2.3 to 3.4 suggests that a slow release and consumption of aqueous Fe(iii) could have taken place during this period. Regardless, most of the leaching agents (aqueous Fe(iii) and H^+^) have been consumed within the first 15 minutes based on potential measurements.

With these observations and theories, it's time to return to the DOE and time dependence when leaching Fe. The lack of reactants after 15 minutes indicates that the system was not kinetically, but more likely mass transport limited. A relevant hypothesis was that transport of the product to the solution was the rate limiting step. This is feasible since FeCl_2_ can be assumed to have been formed rapidly with limited time to diffuse out. Hydrolysis products formed simultaneously near the swarf surface could then have formed a protective structure that inhibited product transport from the swarf surface into the aqueous phase. With the gradual pH increase seen in Fig. 4b, this structure could have been deteriorated and FeCl_2_ dissolved which explains the time dependence of % *E*_s,Fe_. This theory also explains why no major change in potentials was observed over time since a smaller increase of [FeCl_2_] in the bulk would not have affected ORP and pH significantly.

**Fig. 5:**
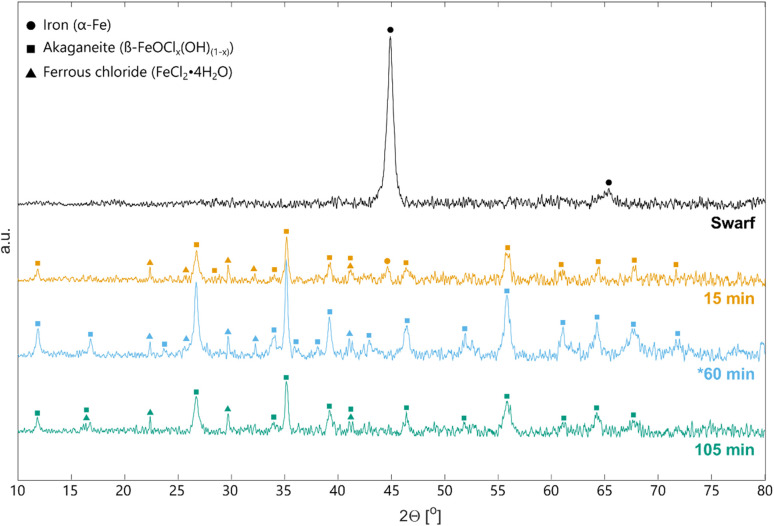
XRD patterns of grinding swarf and leaching residues from DOE experiments 1 (15 min), 3 (105 min) and 16 (*60 min). In all three experiments L/S = 6 mL g^−1^. * Experiment 16 was conducted at 40 °C contrary to 1 and 3 which were conducted at 20 °C.

The proposed hypothesis was difficult to prove without *in situ* analysis of the solid phase, but investigation of leaching residues could at least prove the presence of FeCl_2_. Fig. 5 shows XRD patterns of untreated swarf and solid residues from DOE experiments 1 (15 minutes, 20 °C), 16 (60 minutes, 40 °C) and 3 (105 minutes, 20 °C), all with L/S = 6 mL g^−1^. The background was high, especially at higher 2*θ* angles due to fluorescence of Fe when using a Cu radiation source.^42^ As a result, no conclusions can be drawn about the presence of minor impurities or quantitative amounts of different phases. What Fig. 5 shows is that solid residues predominantly consisted of akaganeite which can have a mixture of OH and Cl incorporate into its lattice.^40^ A minor amount of metallic Fe with a peak at 44.6° was also left after leaching for 15 minutes but disappeared given a longer leaching time. There was also evidence of hydrated FeCl_2_ though its peak size varied relative to akaganeite. Any FeCl_2_ in the residue could have originated either from reaction products which were not dissolved during leaching, or crystallization of dissolved FeCl_2_ from leachate remaining in the filter cake. The latter is probable since final Fe concentrations in the aqueous phase were around 200 g L^−1^ and possibly not reduced sufficiently when washing filter cakes. It was impossible to distinguish between these two FeCl_2_ sources and furthermore, there is a risk of spontaneous FeCl_2_ oxidation (Δ*G*° = −360.1 kJ mol^−1^) and FeO(OH) formation when drying the filter cake in air *via*eqn (15).^43^

15 4FeCl_2_ + 6H_2_O + O_2_ ⇌ 4FeO(OH) + 8HCl

At best, it can therefore be concluded that some amount of FeCl_2_ product remained in the filter cake but the hypothesis about mass transport limitations during leaching is inconclusive.

### 4.4 Comparison of FeCl_3_ with HCl as a leaching agent for grinding swarf

**Fig. 6:**
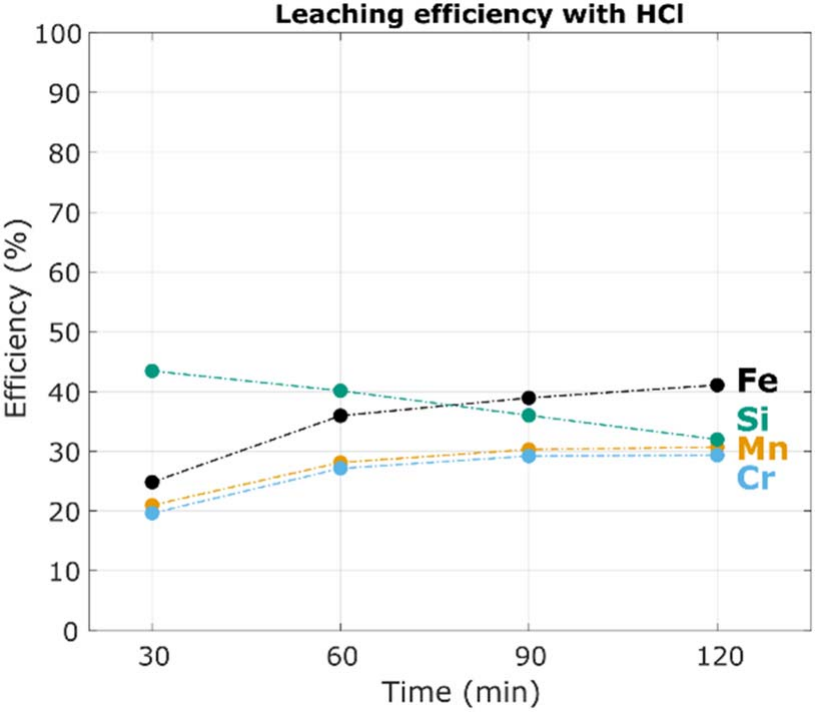
Leaching efficiencies (% *E*_s,M_) for grinding swarf (60.40% Fe, 0.80% Mn, 0.16% Cr, 0.17% Si) in 34% HCl at 60 °C with a liquid to solid ratio (L/S) of 3 mL g^−1^.

In previous work HCl was used as a leaching agent,^11^ and it was of interest to compare the performance of HCl and FeCl_3_ for leaching of steel swarf. Thus far it has been shown that extraction with FeCl_3_ was fast and achieved high efficiencies around 95% under the right conditions. The reaction was completed within 10 minutes although it took longer for the FeCl_2_ product to leach out of the solids completely. For comparison, the same type of swarf but from a different batch was leached with stoichiometric amounts of concentrated HCl (2 : 1 HCl : Fe) instead of FeCl_3_ and efficiencies for Fe, Mn, Cr and Si over time are given in Fig. 6.

Only 41% of Fe and 30% of Mn and Cr were dissolved after 2 h and further leaching was difficult due to slow kinetics despite highly acidic conditions (pH < 0) throughout the testing. This was attributed to the cutting fluids and particularly corrosion inhibitors which protect the steel surface from HCl.^11^ These molecules form protective monolayers by physical and/or chemical adsorption to a surface which separates it from the corrosive environment.^44^ Inhibitors normally contain heteroatoms such as N, S, O and P and conjugated π-bonds and/or polar groups for good adsorption. The coverage and efficiency of the inhibitor are specific to the structure of the molecule and polarity of the surface and external environment. Elevated temperatures have a detrimental effect on protectiveness since it increases corrosion rate and raises the kinetic energy of the inhibitor which decreases adsorption to the steel surface.^45^ In strongly acidic media it can also lead to catalysed rearrangement and fragmentation of the organic molecule whereby it can lose its protective qualities.

Swarf studied in this work contained lubricant with a mixture of mineral oil, amines, carboxylic acids, alkanolamines and heterocyclic compounds according to a material data sheet. This mixture clearly provided good protection against concentrated HCl according to Fig. 6 and the bulk of the swarf was still visually intact. No such protective effects were however observed when leaching with FeCl_3_ which had no problem dissolving the metallic steel completely according to XRDs of leaching residues Fig. 5. Relating this to the adsorption mechanism of the inhibitors, there are several ways in which the FeCl_3_ could have circumvented the protective layer. Firstly, high local temperatures near the surface during reaction could have decreased the adsorption efficiency and surface coverage. In combination with the strong oxidative and acidic environment, this could also have led to decomposition of the inhibitor molecules.^45^ Secondly, it was also probable that the Fe(iii) ion's strong hydrolytic nature and potential to form chloro-complexes created a different hydrodynamic and electrostatic environment compared to the HCl/Fe(ii) mixture.^28,30^ This change in external environment polarity can affect the adsorption strength of the organic molecule to the metal surface.^44^

One final note on Fig. 6 is that the measured silicon concentration was initially high and decreased during leaching. A similar behaviour has been observed when leaching swarf containing colloidal Al_2_O_3_ abrasive particles and suggests that part of the silicon was present as colloidal silica from either the lubricant or abrasive wheel.

### 4.5 Further improvements and recycling process conceptualization

From an engineering perspective, the large temperature increase observed when adding swarf directly to concentrated FeCl_3_ would be difficult to control and unacceptable on an industrial scale. A different approach can be to dose the FeCl_3_ into a mixture of swarf, water and FeCl_2_ in a controlled manner. Based on the study of redox potentials in Fe(iii) and Fe(ii) mixtures in Fig. 2, potentiostatic leaching at a controlled ORP should be feasible since even small amounts of Fe(iii) contributed to creating an oxidative environment for leaching Fe. This method has the advantage that only the necessary amount of reagent is added with minimal excess which is ideal for heterogeneous materials such as grinding swarf.^46^ Another important engineering consideration is selection of process equipment materials. Chloride solutions are corrosive and FeCl_3_ solutions are well known to dissolve stainless steel.^47^ Common practice in coagulant production is therefore to use glass or rubber lined reactors but other more expensive chloride resistant materials such as tantalum can also be used.^48^

When designing a recycling process flowsheet, purification of the FeCl_2_ solution can be achieved by addition of another small amount of swarf to consuming excess Fe(iii) and H^+^. This precipitates lubricant oils and alloying elements such as Al, Cr, and Mo by hydrolysis, and Co, Cu and Ni by cementation.^11^ These impurities can then be filtered out and collected as a solid byproduct while the FeCl_2_ solution can be re-oxidised to FeCl_3_*via e.g.* chlorine oxidation, but preferable safer techniques such as pressure oxidation with O_2_ and HCl, or electrochemistry.^49–51^ Part of the FeCl_3_ can then be recycled to process more swarf and the excess sold as product.

In terms of scalability one of the major challenges is collection and transport of grinding swarf to a centralised recycling plant. The waste quantities available are vast but spread across a multitude of workshops which necessitates sophisticated logistics, especially due to the swarf's flammability.^5^ Ideally the site should be located near a hydrochloric acid or other chloride source to reduce the transport of reagents as these represent the majority of process input material. On the product end, the demand for iron chloride solutions is currently a bottleneck for recycling of grinding swarf which also limits the scalability of this method. Although there is much room for replacing virgin iron ore as an input material for these chemicals, the availability of iron in grinding swarf exceeds the need in dominant areas such as water treatment. There are however several indicators that the iron chloride demand in sustainability applications may grow in the near future due to new wastewater treatment regulations, increase in biogas production, and a growing demand for high purity iron sources in lithium iron phosphate (LFP) batteries.^14,15,52^

## 5 Conclusion

The purpose of this work was to investigate recycling of grinding swarf and more specifically, a method for producing FeCl_2_ by dissolution metals in FeCl_3_ as an alternative to leaching with HCl. It was found that 95% of Fe in the swarf could be solubilized within 1 h using FeCl_3_ as a leaching agent compared to only 40% after 2 h with HCl. The effectiveness of the Fe(iii) ions was partly because of fast reaction kinetics with metallic Fe but also due to its ability to circumvent or decompose corrosion inhibitors in the cutting fluids covering the swarf surface. While the good reactivity between swarf and FeCl_3_ was positive, the reaction was also highly exothermic and addition of swarf to a concentrated solution resulted in significant heat development and hydrolysis. Rapid formation of FeCl_2_ and solid hydrolysis products near the swarf surface were found to trap and kinetically limit transport of FeCl_2_ into the solution. Most of the product was however dissolved eventually by partial redissolution of the protective hydroxides and by leaching out. The exothermic reaction can be more difficult to control in larger scale and side reactions between Fe and H^+^ were likely to result in some undesirable H_2_ formation and loss of reactant. Therefore, a method where FeCl_3_ is slowly dosed into a mixture of swarf and aqueous FeCl_2_ based on redox control was proposed as an improvement of the current method. This should be feasible since addition of even small amounts of Fe(iii) in a solution of Fe(ii) was found to produce oxidizing conditions suitable for leaching Fe. The findings in this work contribute to understanding the dissolution mechanisms involved when leaching steel scrap with Fe(iii). This lays the foundation for development of an efficient and safe hydrometallurgical recycling process for grinding swarf with minimal need for H_2_ handling. The many potential future sustainability applications of iron chloride in water treatment, biogas purification, and LFP production can also make this recycling alternative more economically attractive and ultimately help reduce incineration and landfilling of hazardous manufacturing waste.

## Author contributions

T. O. and F. G. were responsible for conceptualization, experimental work, data curation and writing of the paper. M. L. was responsible for leaching swarf with HCl and reviewing the manuscript. D. C. R. E. and M. P. were responsible for funding acquisition, supervision and review of the manuscript.

## Conflicts of interest

T. O. reports a relationship with Anferra AB as a board member, shareholder and inventor of a pending patent owned by the company. M. L. reports a relationship with Anferra AB as an employee, board member and shareholder. Other authors declare that they have no known competing financial interests or personal relationships that could have appeared to influence the work reported in this paper.

## Supplementary Material

RA-015-D5RA06768E-s001

## Data Availability

Relevant data supporting this article have been presented or included as part of the supplementary information (SI). Additional raw data files and software will be shared upon request. See DOI: https://doi.org/10.1039/d5ra06768e.
