# Supplementary material for: Hydrometallurgical recycling of steel grinding swarf via oxidative leaching using ferric chloride
Source: RSC Adv. 2025 Oct 24;15(48):40675–86. doi: 10.1039/d5ra06768e (PMC12551445; doi:10.1039/d5ra06768e)
Supplement: RA-015-D5RA06768E-s001 [file RA-015-D5RA06768E-s001.pdf]

Supplementary Information (SI) for RSC Advances.
This journal is © The Royal Society of Chemistry 2025

Supplementary material

Frost diagram for Fe

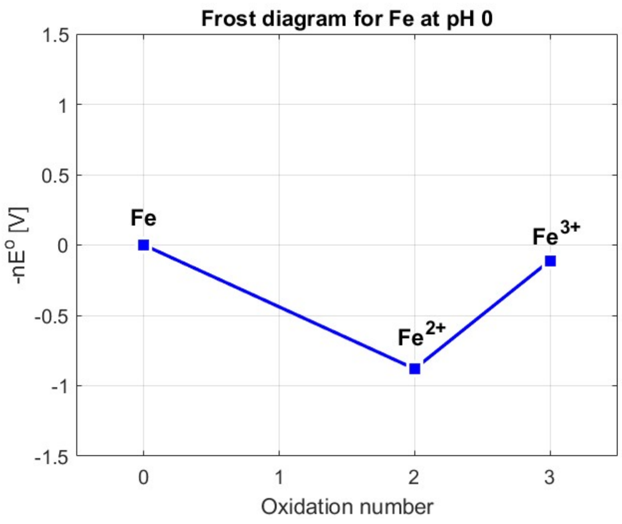

Figure S1: Frost diagram for Fe showing the relative stability of compounds under acidic conditions.

Experimental setup

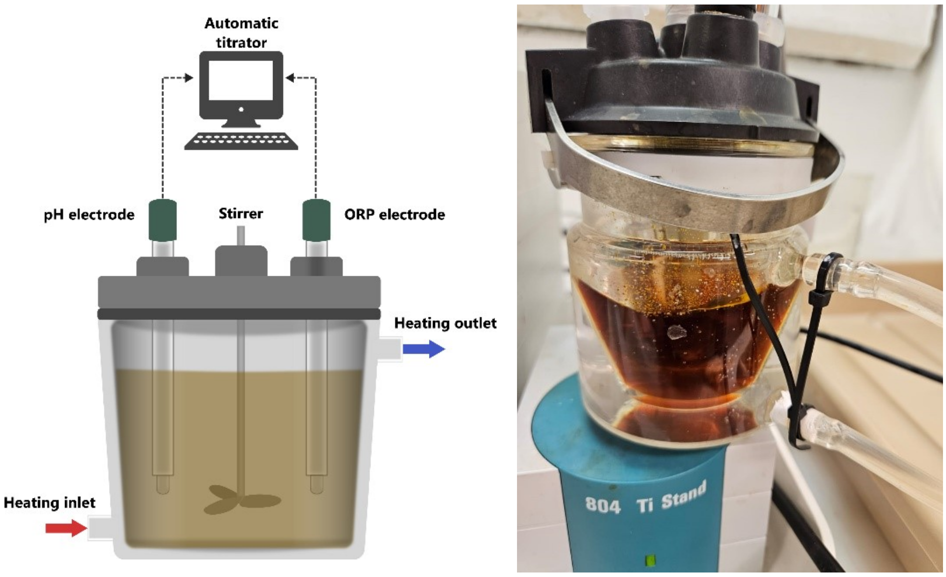

Figure S2: Schematic view (left) of the experimental setup and picture (right) of the leaching reactor.

Iron speciation by complexation and UV/vis spectroscopy

Diluted leachates with Fe concentrations between 1-3 ppm and volumes of 5 mL were transferred to two separate test tubes. To each vial, 3 mL of ammonium acetate buffer (pH 4.75, Sigma Aldrich) and 1 mL of aqueous 1 g/L 1,10-phenanthroline (≥99%, Sigma Aldrich) were added. Phenanthroline reacts specifically with Fe(II) between pH 2-9 to form an orange- red complex that can be measured by UV-Vis spectroscopy. One vial also received 1 mL of 100 g/L hydroxylamine hydrochloride (≥99%, Merck)

reducing agent while the other received 1 mL water. With the reducing agent, all iron is reduced to Fe(II) which complexes with phenanthroline whilst with water, only Fe(II) formed during leaching complexes. The solutions were allowed to stand for 10 minutes for colour development before analysis with UV/vis spectroscopy at 510 nm in plastic cuvettes. Total iron concentrations were determined using the tube with reducing agent, while Fe(II) concentrations were obtained from the other vial. The concentration of Fe(III) could then be calculated as the difference between the two values.

Reference calibration standards containing 0.25-5 ppm Fe were prepared from a 1000 ppm iron standard solution using the described method with reducing agent to correlate experimental readings with iron concentrations. The extinction coefficient was determined to be ε=10993 $cm^{-1}M^{-1}$ which was in good agreement with literature values (Hayes et al., 2011; Mehlig and Hulett, 1942).

When applying the method in practice to estimate [Fe(III)], it was found that the variability in determination of Fe(II) and total Fe was relatively high. This sometimes led to [Fe(II)] estimates that were significantly higher than the total [Fe] and consequently negative [Fe(III)]. Another observation was that the total [Fe] estimates in some cases deviated from the relatively sound ICP-OES results and predicted leaching efficiencies >100%. The reason for this was unclear but could have been caused by the many steps involved in sample preparation. This needed to be done directly after leaching and filtration to ensure minimum oxidation of the Fe(II) and a high variability in the experimental procedure itself was thus likely.

Regression model optimization

Table S1: Regression model optimization for Fe where least significant model parameters were removed stepwise until a maximum R^2_{adj} value was achieved.

Parameter removed	F_R	$F_{crit,R}$	F_{LOF}	$F_{crit,LOF}$	R^2	R^2_{adj}	σ
-	4.82	3.68	3.21	19.30	0.861	0.683	12.49
x_1x_3	6.19	3.44	2.68	19.33	0.861	0.722	11.69
x_1	7.91	3.29	2.31	19.35	0.860	0.751	11.05
x_1x_2	9.69	3.22	2.14	19.37	0.853	0.765	10.75
x_1^2	10.87	3.20	2.21	19.38	0.832	0.755	10.97

References

Hayes, W.A., Mills, D.S., Neville, R.F., Kiddie, J., Collins, L.M., 2011. Determination of the molar extinction coefficient for the ferric reducing/antioxidant power assay. Anal Biochem 416, 202–205. https://doi.org/10.1016/J.AB.2011.05.031

Mehlig, J.P., Hulett, H.R., 1942. Spectrophotometric Determination of Iron With o-Phenanthroline and with Nitro-o-phenanthroline. Industrial and Engineering Chemistry - Analytical Edition 14, 869–871. https://doi.org/10.1021/I560111A018/ASSET/I560111A018.FP.PNG_V03
